# Exploring reciprocal causation: bidirectional mendelian randomization study of gut microbiota composition and thyroid cancer

**DOI:** 10.1007/s00432-023-05535-y

**Published:** 2024-02-03

**Authors:** Jiating Zhou, Xuan Zhang, Zilan Xie, Zhi Li

**Affiliations:** 1 Department of Clinical Pharmacology, Xiangya Hospital, Central South University, 87 Xiangya Road, Changsha, 410008 Hunan People’s Republic of China; 2 Institute of Clinical Pharmacology, Central South University and Hunan Key Laboratory of Pharmacogenetics, 110 Xiangya Road, Changsha, 410078 People’s Republic of China; 3 Engineering Research Center of Applied Technology of Pharmacogenomics, Ministry of Education, 110 Xiangya Road, Changsha, 410078 People’s Republic of China; 4 National Clinical Research Center for Geriatric Disorders, 87 Xiangya Road, Changsha, 410008 Hunan People’s Republic of China; 5 The Second People’s Hospital of Hunan, The General Surgery Department, No.427, Section 3, Furong Middle Road, Changsha, 410078 People’s Republic of China

**Keywords:** Gut microbiota, Thyroid cancer, Mendelian randomization, Causal relationship, Instrumental variables

## Abstract

**Background:**

While an association between gut microbiota composition and thyroid cancer (TC) has been observed, the directionality and causality of this relationship remain unclear.

**Methods:**

We conducted a bidirectional two-sample Mendelian randomization (MR) analysis to investigate the causal effect between gut microbiota composition and TC. Gut microbiota data were derived from a diverse population encompassing various ethnicities (*n* = 18,340 samples), while TC data were sourced from an European population (*n* = 218,792 samples). Instrumental variables, represented by single nucleotide polymorphisms (SNPs), were employed to assess the causal relationship using multiple MR methods, including inverse-variance weighting (IVW), weighted median, weighted mode, MR-Egger, and simple mode. F-statistics and sensitivity analyses were performed to evaluate the robustness of the findings.

**Results:**

Our investigation identified a comprehensive set of 2934 instrumental variables significantly linked to gut microbiota composition (*p* < 1 × 10^-^^5^). The analysis illuminated notable candidates within the phylum Euryarchaeota, including families Christensenellaceae and Victivallaceae, and genera Methanobrevibacter, Ruminococcus2, and Subdoligranulum, which emerged as potential risk factors for TC. On the other hand, a protective influence against TC was attributed to class Betaproteobacteria, family FamilyXI, and genera Anaerofilum, Odoribacter, and Sutterella, alongside order Burkholderiales. Further enhancing our insights, the integration of 7 instrumental variables from TC data (*p* < 1 × 10^-^^5^) disclosed the regulatory potential of one family and five genera. Notably, the genus Coprobacter innocuum group (*p* = 0.012, OR = 0.944) exhibited the highest probability of regulation. Our meticulous analyses remained free from significant bias, heterogeneity, or horizontal pleiotropy concerns.

**Conclusion:**

Through a bidirectional two-sample Mendelian randomization approach, we elucidated a potential bidirectional causal relationship between gut microbiota composition and TC. Specific microbial taxa were associated with an increased risk or conferred protection against TC. These findings advance our understanding of the complex interplay between the gut microbiota and TC pathogenesis, offering new insights into the therapeutic potential of modulating the gut microbiota for managing TC.

**Supplementary Information:**

The online version contains supplementary material available at 10.1007/s00432-023-05535-y.

## Introduction

Thyroid Cancer (TC) is the most common endocrine malignancy globally, with its incidence steadily rising over the past few decades (Siegel et al. 2023). While the etiology of TC is multifactorial, known risk factors include radiation exposure, genetics, and environmental factors. However, the precise mechanisms underlying TC development and progression remain incompletely understood (Vaccarella and Maso 2021). In recent years, emerging evidence suggests a potential connection between the gut microbiota and TC, highlighting the role of the gut-thyroid axis in the pathogenesis of TC.

Thyroid development derives from primitive gut cells, and both gastric mucosal cells and thyroid follicular cells share a common embryonic origin, suggesting the potential for microbial colonization in the thyroid (Cellini et al. 2017). Any thyroid disorder is closely associated with thyroid hormone levels or function, which in turn can impact the composition of the gut microbiota (Virili and Centanni 2017). Functional thyroid disorders are associated with excessive bacterial growth and distinct microbial composition (Bargiel et al. 2021). Simultaneously, gut microbiota can exert an influence through the gut-brain axis, integrating immune, metabolic, and endocrine signals both peripherally and centrally (Jašarević et al. 2016). The gut microbiota constitutes a complex ecosystem of microorganisms within the gastrointestinal tract, playing a crucial role in various aspects of human health, including metabolism, immune regulation, and the interactions between the host and microorganisms (Hou et al. 2022). Numerous studies have demonstrated a close relationship between gut microbiota and the occurrence and progression of gastrointestinal tumors (Tong et al. 2021). With the progression of research, researchers have progressively illuminated the substantial roles that gut microbiota assume in the progression and management of extraintestinal tumors (Bishehsari et al. 2020; Matson et al. 2021; Park et al. 2022). These encompass liver cancer, pancreatic cancer, melanoma, hematologic malignancies, and breast cancer. Research indicates that the contributions of the gut microbiota to carcinogenesis can be categorized into two major classes. The first class involves DNA damage and cell apoptosis, where organisms like Escherichia coli and Bacteroides fragilis might impact the stability of the host genome, leading to mutational events, disruption of host DNA, and the initiation of colorectal carcinogenesis (Arthur et al. 2014). The second class involves modulating inflammatory responses, with many microbiota communities associated with cancer activating pattern recognition receptors such as Toll-like receptors, subsequently triggering the activation of nuclear factor Kappa B through signal transduction in the tumor microenvironment (Kostic et al. 2013). Significantly, within the realm of TC, Several clinical studies have noted significant variations in the gut microbiota composition of thyroid cancer patients when compared to that of healthy individuals. Feng et al. found that TC patients had higher Firmicutes and Proteobacteria proportions, and lower Bacteroidetes levels compared to healthy controls (Feng et al. 2019). In another clinical study, Zhang found differing gut microbiota composition between TC patients and healthy controls, correlating gut microbiota with thyroid-stimulating hormone (TSH) and free triiodothyronine (FT3) levels in TC patients (Zhang et al. 2019). Nevertheless, in the realm of basic medical research, there remains an absence of substantiated evidence to establish a connection between TC and the composition of gut microbiota.

Although some observational epidemiological studies have suggested a connection between gut microbiota and TC, confirming a causal link between them through observational research is challenging due to potential confounding factors and reverse causation. Establishing a bidirectional causal relationship between gut microbiota and TC not only aids in unraveling underlying mechanisms but also elucidates potential therapeutic targets. Mendelian randomization (MR) is a powerful analytical method that employs genetic variations as instrumental variables to deduce causal relationships between exposures and outcomes (Emdin et al. 2017). Utilizing genetic variations closely tied to the exposure of interest, MR analysis offers valuable evidence for causality, mitigating limitations inherent in observational studies susceptible to confounding and reverse causation (Chen et al. 2022; Tin and Köttgen 2021). In the context of the gut microbiota and TC, the application of a bidirectional two-sample Mendelian randomization study can elucidate the potential causal connection between gut microbiota composition and TC risk.

Hence, in this study, we conducted a bidirectional two-sample Mendelian randomization analysis to investigate the causal relationship between gut microbiota composition and TC risk. Leveraging publicly available summary datasets from genome-wide association studies (GWAS) for gut microbiota composition and TC, we identified genetic variations as instrumental variables. By employing various MR methods, including inverse variance-weighted (IVW), weighted median, weighted mode, MR Egger, and simple mode, our aim was to robustly assess the causal impact between gut microbiota and TC risk.

## Materials and methods

### Study design and the assumption of MR

**Fig. 1:**
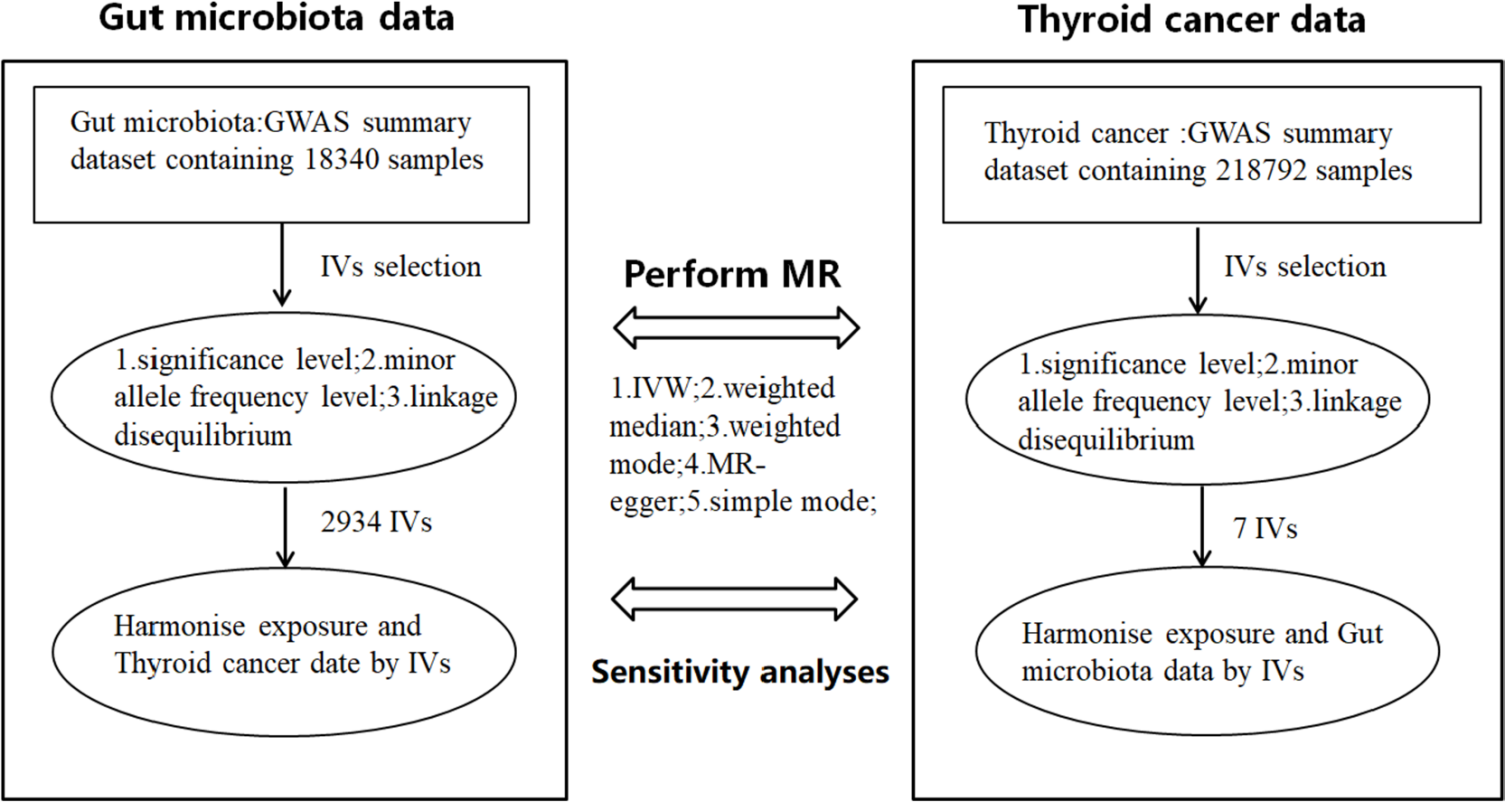
The workflow of MR analysis is as shown in the diagram. In this diagram, two distinct components are highlighted: the examination of gut microbiota exposure and the examination of thyroid cancer (TC) exposure, both conducted independently. The diagram visually represents the steps taken to investigate the causal relationships between these exposures and their respective outcomes. *GWAS* Genome-Wide Association Study, *IV* Instrumental Variable, *IVW* Inverse-Variance Weighting, *MR* Mendelian Randomization

The depicted flowchart succinctly illustrates the holistic procedure outlined in Fig. 1. To comprehensively investigate the bidirectional causal association between gut microbiota composition and TC, an in-depth bidirectional two-sample Mendelian randomization (MR) analysis was undertaken. This analytical approach harnessed aggregated statistical data from genome-wide association studies (GWAS). The MR framework employs genetic variations as instrumental variables (IVs) to rigorously quantify the causal impact connecting the exposure and the outcome. Building upon the foundational principles expounded by Bowden and colleagues, the two-sample MR analysis rests on the following core assumptions (Bowden et al. 2015): (1) the selected IVs manifest an inherent link with the exposure; (2) the IVs remain unaffected by any latent confounding factors that might skew the intricate interplay between exposure and outcome; and (3) the IVs singularly exert influence on the outcome (TC) exclusively through the exposure, bypassing alternative pathways.

### Data sources

The data for this study was derived from two distinct GWAS summary datasets: one focused on gut microbiota composition and the other centered around TC. The first dataset delved into the intricacies of gut microbiota composition and drew upon samples from a wide spectrum of 24 cohorts representing various ethnicities, including European, Middle Eastern, East Asian, American Hispanic/Latin, and African American populations. In total, this compilation encompassed data from 18,340 individuals (Kurilshikov et al. 2021). Stringent measures were undertaken to eliminate any potential batch effects, and a comprehensive breakdown of the individual cohorts can be referenced in a previous publication (Kurilshikov et al. 2021). When investigating the composition of the gut microbiota, particular attention was given to several hypervariable regions within the 16S ribosomal RNA (rRNA) gene, namely V1–V2, V3–V4, and V4. Taxonomic profiling was approached by leveraging the Ribosomal Database Project (RDP) classifier (version 2.12) to align reads with the SILVA reference database. Prior to this alignment, the samples underwent a process of rarefaction to achieve a standardized read count of 10,000 reads, employing a predefined random seed. Consequently, a refined selection of 211 taxa met the threshold for taxon inclusion, with the cut-off points determined at a posterior probability of 0.8. This inclusive selection comprised taxa spanning across nine phyla, 16 classes, 20 orders, 35 families, and 131 genera. It is pertinent to underscore that unidentified taxa were meticulously excluded from the analysis, ensuring the precision of the results.

The genetic association data summary was sourced from the European population thyroid cancer dataset in the Genome-Wide Association Studies (GWAS) domain (https://GWAS.mrcieu.ac.uk/datasets/finn-b-c3_thyroid_gland/). The GWAS derived from the FinnGen study encompasses 989 cases of thyroid cancer and 217,803 control subjects, constituting a dataset of 16,380,466 single nucleotide polymorphisms (SNPs). The FinnGen research undertook a comprehensive GWAS meta-analysis across 13 cohorts and biobanks on a nationwide scale within Finland.

### Instrumental variable selection

To identify suitable instrumental variables (IVs) that could potentially indicate underlying associations between gut microbiota composition and TC, distinct thresholds were applied, tailored to the characteristics of the exposure. We meticulously extracted all available data from the comprehensive summary statistics of the gut microbiota GWAS, encompassing traits across various taxonomic levels including phylum, class, order, family, and genus, with these traits being marked by their relative abundance (RA) metrics. To establish the gut microbiota as the exposure of interest, a strategic approach was adopted, involving the selection of single nucleotide polymorphisms (SNPs) that demonstrated significant associations with TC. This selection process adhered to the SNP association threshold previously validated by Sanna et al. (2019), set at a stringent value of 1.0 × 10^–5^. To effectively address the potential influence of linkage disequilibrium (LD) patterns, a meticulous clumping strategy was executed on SNPs within each distinct feature, utilizing the PLINK software (v1.9) (Purcell et al. 2007). This clumping procedure aimed to retain exclusively independent SNPs, safeguarding the precision of subsequent analyses. As a mechanism to account for LD-related intricacies, the LD threshold was conservatively set at *r*^2^ < 0.1, ensuring the robustness of the retained SNPs. Furthermore, the clumping window, an essential parameter in this context, was meticulously defined at a range of 500 kb. The estimation of LD patterns was carried out with meticulous reliance on sequencing data sourced from the third phase of the globally recognized 1000 Genomes Project.

On the contrary, when TC was considered as the exposure, the selection of instrumental variables (IVs) was based on significant genome-wide statistical thresholds (*p* < 1 × 10^–5^). A linkage disequilibrium (LD) threshold of 0.001 and a clumping window of 10,000 kb were employed. All other parameters remained consistent with those applied in the analysis of gut microbiota composition.

The F-statistic serves as a crucial indicator in MR assessments to ascertain whether weak instrumental variables (IVs) are prone to confounding. The strength of the correlation between the SNP locus and the exposure factor is evaluated through the *F*-value associated with each SNP. Typically, when *F* > 10, the presence of bias in the instrumental variable is considered negligible, leading to the exclusion of SNP loci with *F* ≤ 10.

### MR analysis

To detect the causal effects between gut microbiota composition and TC, we employed five commonly used MR methods: inverse-variance weighting (IVW), weighted median, weighted mode, MR-Egger, and simple mode. These methods estimate the causal effect by combining the ratio estimates for each SNP, weighted regression of SNP-outcome effects on SNP-exposure effects, or unweighted mode of the empirical density function of causal estimation (29–33). The IVW method provides a weighted regression estimate of the causal effect by combining SNP-specific estimates, while the weighted median method provides unbiased estimates even if up to 50% of the information comes from invalid IVs. The weighted mode method is consistent even with invalid IVs when the largest number of similar individual instrument causal effect estimates comes from valid instruments. MR-Egger regression provides a causal estimate and can detect small study bias, and the simple mode is an unweighted mode of the causal estimation distribution (Burgess et al. 2013; Bowden et al. 2016; Hartwig et al. 2017). The Wald ratio was used when only one IV was available from the exposure. The findings are primarily grounded in the IVW method, with the other four methods serving as supplementary analyses (Boehm and Zhou 2022). When the direction of causality remains consistent across these five approaches, it is considered a relatively stable causal association. The causal effect was expressed as an odds ratio (OR) when the *p*-value was < 0.05 based on the MR analysis (Hu et al. 2022).

To evaluate the robustness and validity of the results, we performed sensitivity analyses. Heterogeneity among the instrumental variables was assessed using Cochran’s Q statistics, with *p* < 0.05 indicating significant heterogeneity (Bowden and Holmes 2019). Horizontal pleiotropy, which suggests IVs are associated with the outcome through pathways other than the exposure, was tested using MR-PRESSO (*p* < 0.05) (Morrison et al. 2020). Leave-one-out analysis was conducted to identify potential outliers by sequentially excluding individual SNPs and assessing their impact on the causal effect estimation using the inverse-variance-weighted method.

All statistical analyses were performed using R software (version 4..0; The R Foundation for Statistical Computing, Vienna, Austria). The main R packages used in this study were Two Sample MR, MRPRESSO, and Mendelian Randomization.

## Results

The bidirectional two-sample Mendelian randomization (MR) analysis revealed potential causal effects between gut microbiota composition and TC. Specifically, we investigated the influence of the gut microbiota on the occurrence of TC and the impact of TC on the composition of the gut microbiota.

### Causal effects of gut microbiota on TC

To comprehensively investigate the potential causal relationships between the gut microbiota and TC, we extensively employed a collection of 2934 IVs. Ensuring the robustness of our approach, the F-statistic for each SNP surpassed the threshold of 10, confirming the absence of any weak instrument bias (Supplementary material 1). This array of IVs spanned a spectrum of taxonomic levels, encompassing five distinct phyla, 16 diverse classes, a solitary order, 29 families, and an array of 115 genera, reflecting a broad taxonomic diversity. The range of IVs employed varied from 1 to 24, allowing us to comprehensively account for diverse genetic variation. By meticulously applying the MR analysis framework, we effectively combined the effects of SNPs originating from the same gut microbiota constituents. Moreover, through the application of the MR methodology, we uncovered 1 phylum, 1 specific class, 1 order, 3 families, and 6 distinct genera that evidently exerted a discernible causal influence on TC, as evidenced and elaborated in the detailed findings presented in Table 1.

**Table 1:** The causal effects of the gut microbiota on individuals with thyroid cancer (TC)

Exposure	Method	Number of SNPs	OR	*P*-value
Class.Betaproteobacteria	Inverse variance weighted	11	0.522075	0.015
Family.Christensenellaceae	Inverse variance weighted	11	1.664362	0.015
Family.Christensenellaceae	Weighted median	11	1.892848	0.033
Family.FamilyXI	Inverse variance weighted	8	0.752928	0.037
Family.FamilyXI	Weighted median	8	0.686181	0.041
Family.Victivallaceae	Inverse variance weighted	13	1.26803	0.042
Genus.Anaerofilum	Inverse variance weighted	11	0.702544	0.021
Genus.Methanobrevibacter	Inverse variance weighted	6	1.504903	0.027
Genus.Odoribacter	Inverse variance weighted	7	0.531732	0.04
Genus.Ruminococcus2	Inverse variance weighted	15	1.846461	0.0016
Genus.Subdoligranulum	Inverse variance weighted	11	1.906763	0.01
Genus.Sutterella	Inverse variance weighted	12	0.59618	0.024
Order.Burkholderiales	Inverse variance weighted	10	0.528774	0.021
Phylum.Euryarchaeota	Inverse variance weighted	12	1.308882	0.029

The exposure represents the specific taxa for the causal effect between the gut microbiota and thyroid cancer (TC); the method is for Mendelian randomization (MR) analysis in each row; the number of single nucleotide polymorphisms (SNPs) is the instrumental variables (IVs) for calculations; and the p-values and odds ratios (ORs) indicate significance and effect size, respectively. *MR* Mendelian randomization, *SNP* single nucleotide polymorphism, *OR* odds ratio

**Fig. 2:**
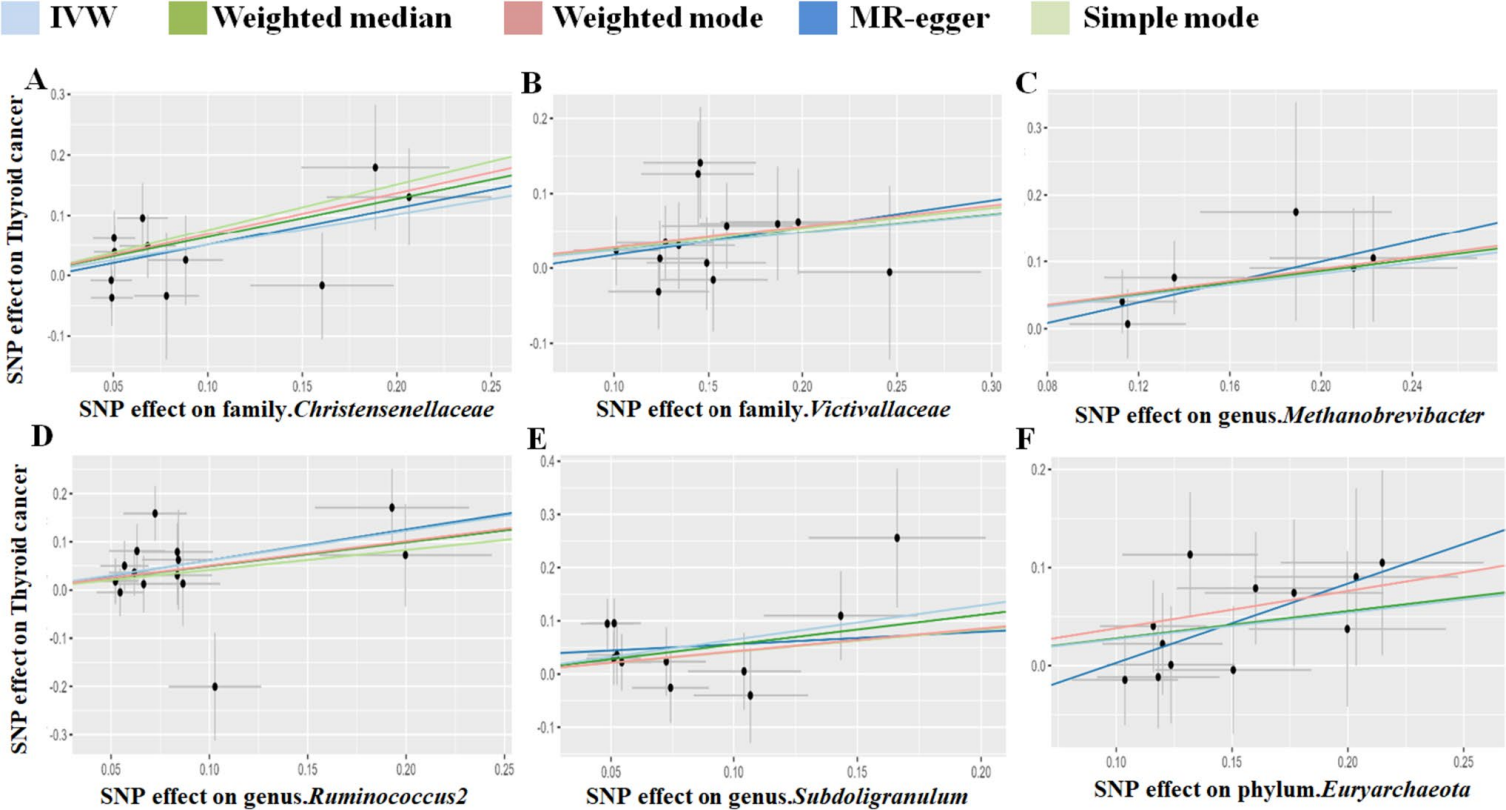
The scatterplot depicts the assessment of the potential risk impact of the gut microbiota on TC using SNPs and five MR methods (A-F). Each dot represents an SNP from the gut microbiota GWAS summary dataset. *x*-axis: SNPs' effect on gut microbiota (position =|β-value|, error bar = SE from gut microbiota GWAS). *y*-axis: SNPs’ effect on TC (position = flipped β-value, error bar = SE from TC GWAS). Line colors: MR techniques (IVW, weighted median, MR Egger, weighted mode, simple mode). Slope = *b*-value from methods, indicating gut microbiota's causal effect on TC. Positive slope: exposure as risk factor; negative: opposite. *TC* thyroid cancer, *MR* Mendelian randomization, *SNP* single nucleotide polymorphism

**Fig. 3:**
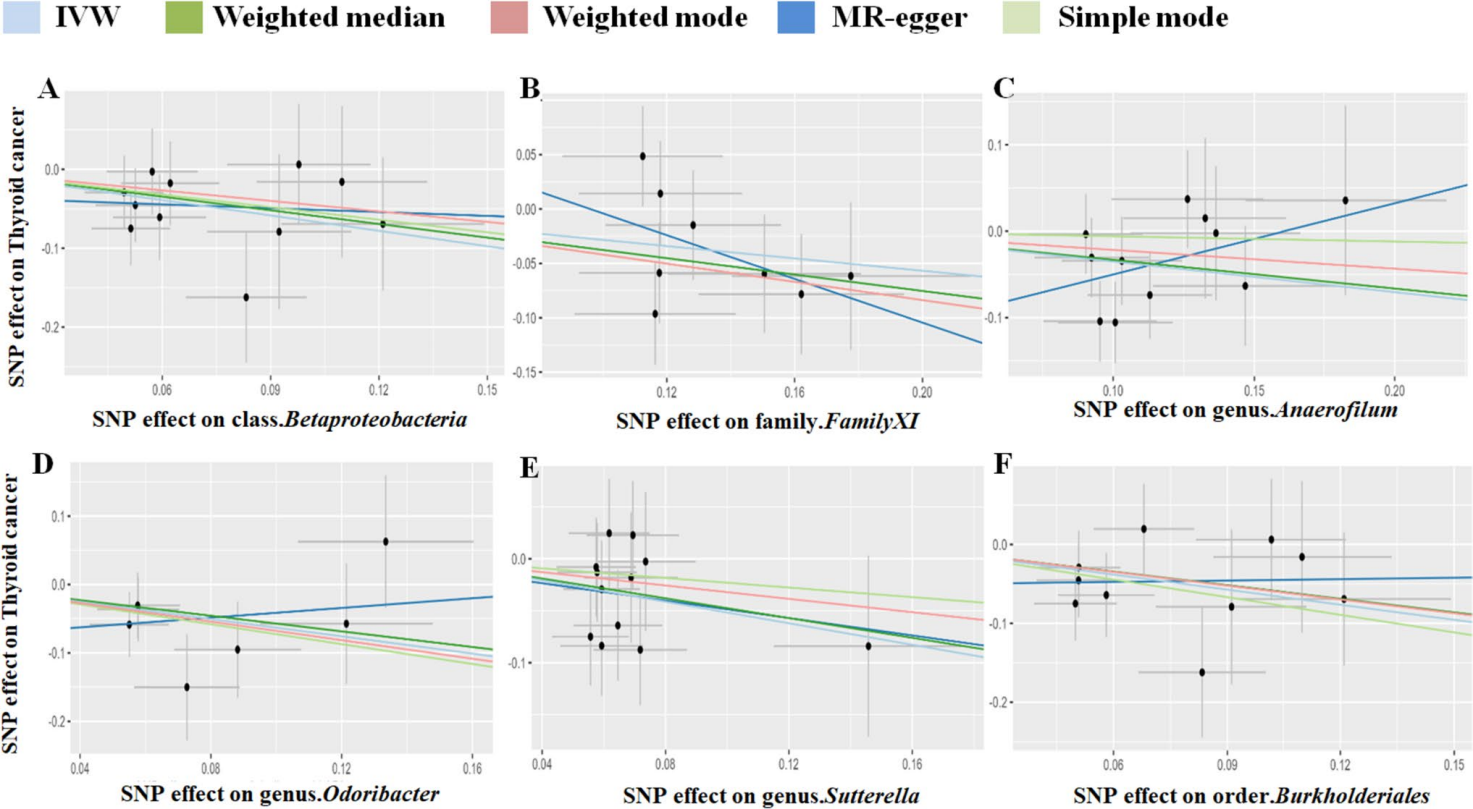
The scatterplot illustrates the evaluation of the potential protective effect of the gut microbiota on thyroid cancer (TC) using single nucleotide polymorphisms (SNPs) and five MR methods (A-F). Each dot represents an SNP from the gut microbiota GWAS summary dataset. *x*-axis: SNPs’ effect on gut microbiota (position =|β-value|, error bar = SE from gut microbiota GWAS). *y*-axis: SNPs’ effect on TC (position = flipped β-value, error bar = SE from TC GWAS). Line colors: MR techniques (IVW, weighted median, MR Egger, weighted mode, simple mode). Slope = *b*-value from methods, indicating gut microbiota’s causal effect on TC. Positive slope: exposure as a risk factor; negative: opposite. *TC* thyroid cancer, *MR* Mendelian randomization, *SNP* single nucleotide polymorphism

Following the MR analysis, we identified several taxonomic groups within the gut microbiota that may play a causal role in TC development. Utilizing the Inverse Variance Weighting (IVW) method, we pinpointed specific taxonomic entities with potential associations with TC risk. Notably, the families Christensenellaceae and Victivallaceae, as well as the genera Methanobrevibacter, Ruminococcus2, and Subdoligranulum within the phylum Euryarchaeota, were implicated as potential risk factors for TC (Fig. 2). Particularly noteworthy, the genus Subdoligranulum exhibited a prominent odds ratio (OR) of 1.907, suggesting its potential significance in TC progression. These findings highlight the potential impact of altered gut microbiota taxonomic groups on TC susceptibility. Conversely, our analysis revealed protective factors, including class Betaproteobacteria, family FamilyXI, and genera Anaerofilum, Odoribacter, and Sutterella, along with order Burkholderiales (Fig. 3). Intriguingly, class Betaproteobacteria demonstrated the strongest protective effect, indicated by its notably low odds ratio (OR = 0.522).

### Causal effects of TC on gut microbiota

**Fig. 4:**
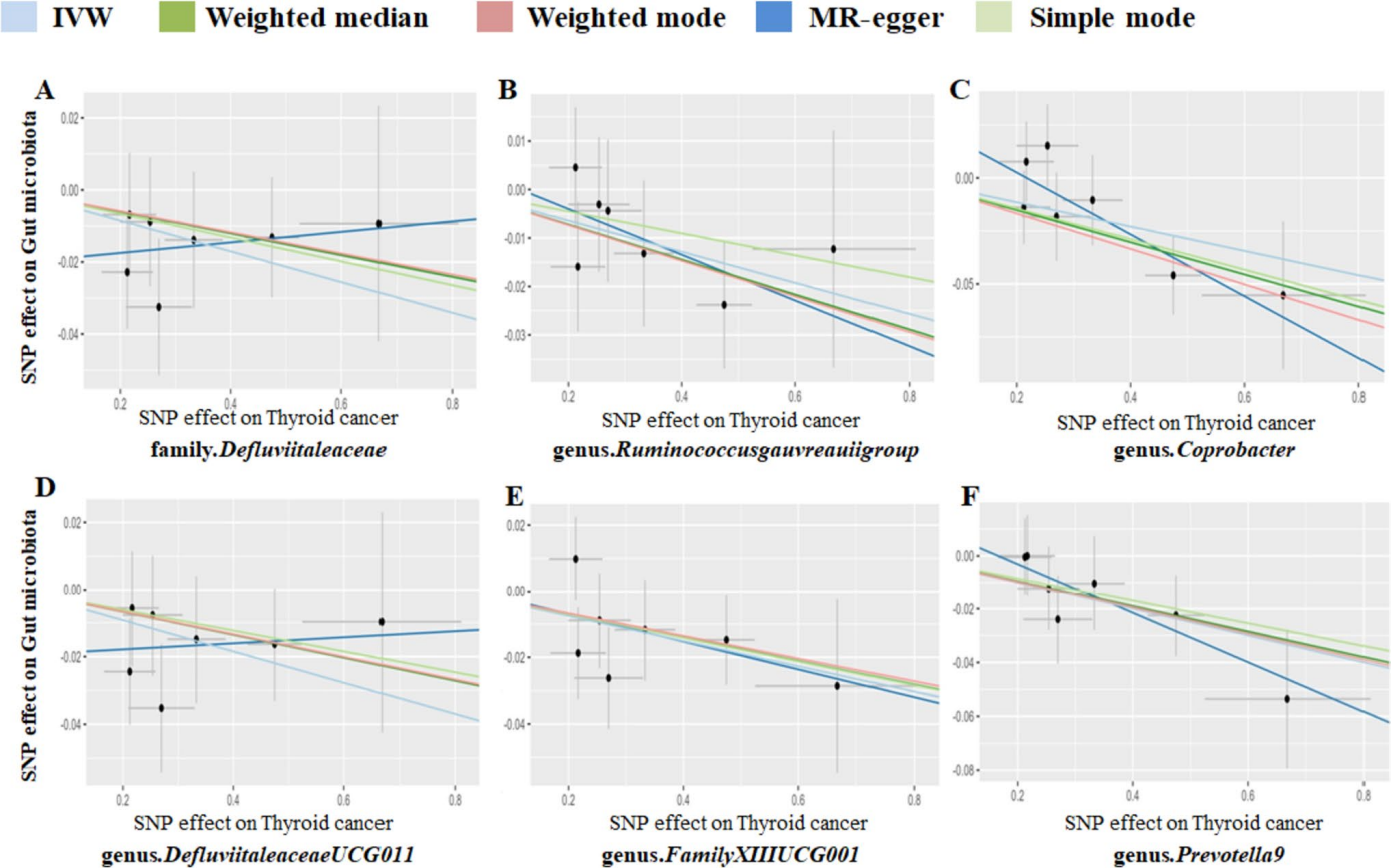
The scatterplot illustrates the assessment of the impact of TC on gut microbiota using SNPs and five MR methods (A-F). Each dot represents an SNP from the gut microbiota GWAS summary dataset. *x*-axis: SNPs’ effect on TC (position =|β-value|, error bar = SE from TC GWAS). *y*-axis: SNPs’ effect on gut microbiota (position = flipped β-value, error bar = SE from gut microbiota GWAS). Line colors: MR techniques (IVW, weighted median, MR Egger, weighted mode, simple mode). Slope = *b*-value from methods, indicating gut microbiota’s causal effect on TC. Positive slope: exposure as risk factor; negative: opposite. *TC* thyroid cancer, *MR* Mendelian randomization, *SNP* single nucleotide polymorphism

In addition to assessing the effects of the gut microbiota on TC, we investigated the influence of TC on the composition of the gut microbiota. During the IV selection, a total of 7 SNPs that met the criteria were identified. The MR analysis revealed that TC has a causal impact on the abundance of one family and five genera within the gut microbiota, as evidenced and elaborated in the detailed findings presented in Table 2. Specifically, following TC onset, it was observed that the abundances of family Defluviitaleaceae, genus Ruminococcus gauvreauii group, genus Coprobacter, genus Defluviitaleaceae UCG011, genus Family XIII UCG001, and genus Prevotella9 were downregulated (Fig. 4). Among these taxonomic groups, the genus Coprobacter exhibited the highest odds ratio (OR) of 0.944, indicating an increased risk associated with the decreased abundance of this genus in TC patients.

**Table 2:** The causal impacts of thyroid cancer (TC) on the composition of the gut microbiota

Outcome	Method	Number of SNPs	*P*-value	OR
Family.Defluviitaleaceae	Inverse variance weighted	7	0.042	0.958259
Genus.Ruminococcusgauvreauiigroup	Inverse variance weighted	7	0.049	0.968421
Genus.Coprobacter	Weighted median	7	0.012	0.926937
Genus.Coprobacter	Inverse variance weighted	7	0.012	0.944251
Genus.DefluviitaleaceaeUCG011	Inverse variance weighted	7	0.028	0.954851
Genus.FamilyXIIIUCG001	Inverse variance weighted	7	0.024	0.962874
Genus.Prevotella9	Inverse variance weighted	7	0.0067	0.951285
Genus.Prevotella9	Weighted median	7	0.046	0.953613

The exposure represents the specific taxa for the causal effect between thyroid cancer (TC) and the gut microbiota; the method is for Mendelian randomization (MR) analysis in each row; the number of single nucleotide polymorphisms (SNPs) is the instrumental variables (IVs) for calculations; and the *p*-values and odds ratios (ORs) indicate significance and effect size, respectively. *MR* Mendelian randomization, *SNP* single nucleotide polymorphism, *OR* odds ratio

### Sensitivity analyses

**Fig. 5:**
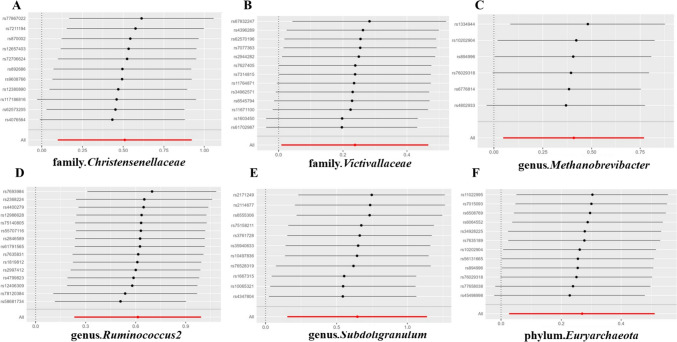
Leave-one-out analysis is used to assess the risk factor impact of gut microbiota on TC. Employing a leave-one-out analysis methodology within the scenarios denoted by (A-F), a comprehensive exploration was conducted to gauge the sensitivity of the risk factor influence attributed to distinct types of gut microbiota on the development of thyroid cancer (TC). The error bar represents the 95% confidence interval with the method of IVW

**Fig. 6:**
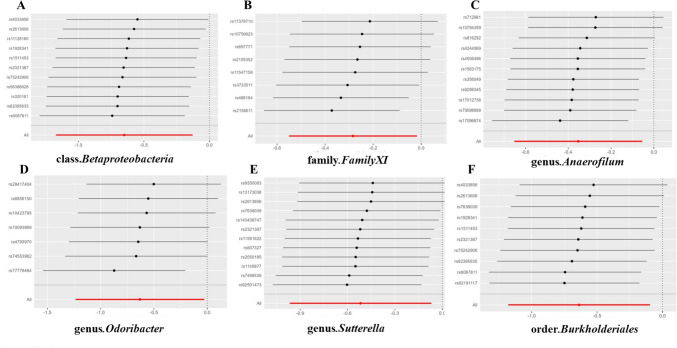
Leave-one-out analysis is used to assess the protective factor impact of gut microbiota on TC. Employing a leave-one-out analysis methodology within the scenarios denoted by (A-F), a comprehensive exploration was conducted to gauge the sensitivity of the protective factor influence attributed to distinct types of gut microbiota on the development of thyroid cancer (TC). The error bar represents the 95% confidence interval using the IVW method

**Fig. 7:**
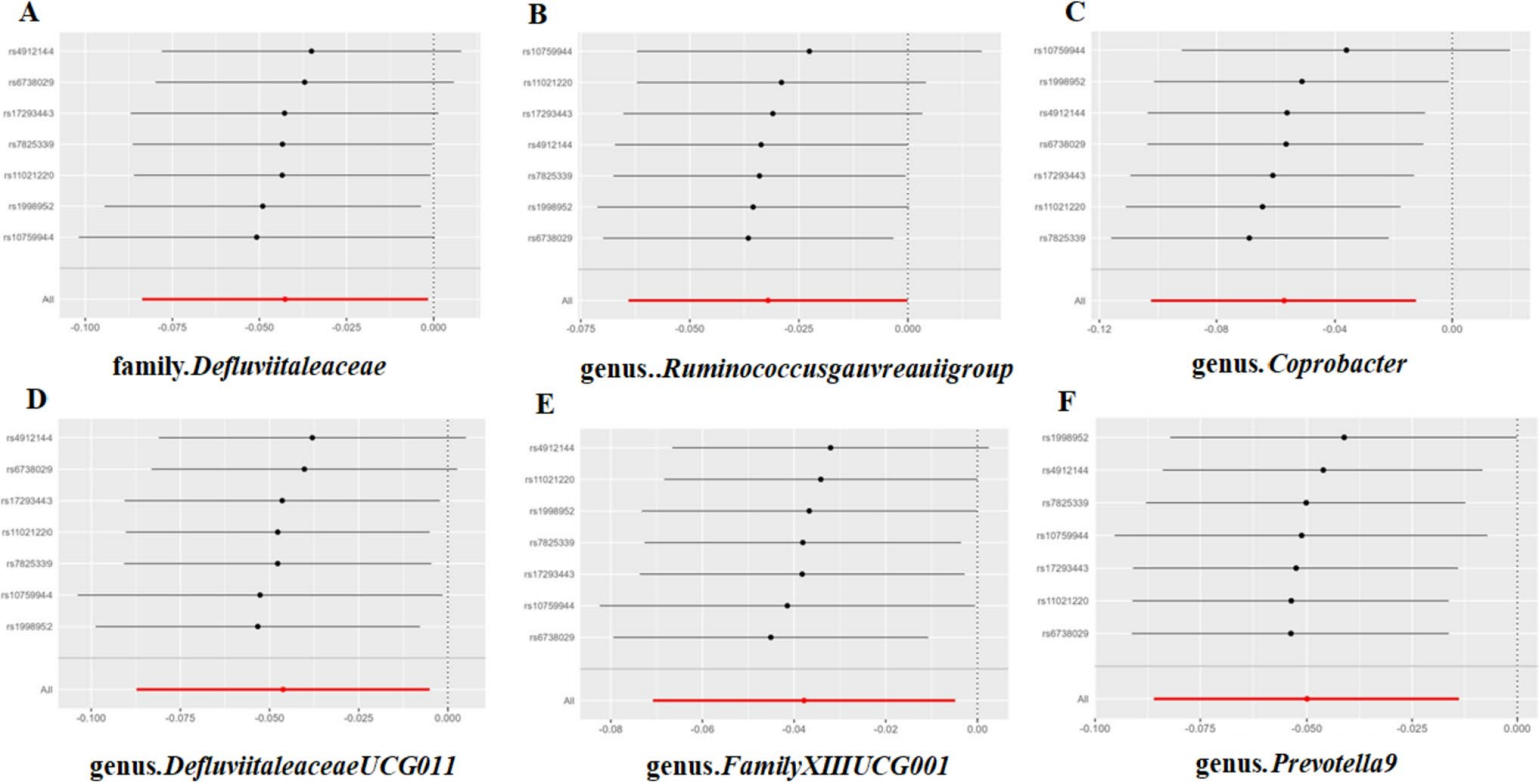
Leave-one-out analysis was employed to investigate the impact of thyroid cancer (TC) on the gut microbiota. Through the utilization of leave-one-out analysis within the contexts outlined by (A-F), a thorough examination was undertaken to assess the sensitivity of the causal effect of TC on various categories of gut microbiota.The error bar represents the 95% confidence interval with the method of IVW

Sensitivity analyses were conducted to assess the robustness of our findings. Heterogeneity statistics, horizontal pleiotropy assessment, and leave-one-out analysis were performed to evaluate the consistency and reliability of the causal effects observed. Heterogeneity analysis revealed no significant evidence of heterogeneity among the investigated variables of the gut microbiota for both the causal effects on TC and the effects of TC on the gut microbiota. Furthermore, we found no evidence of horizontal pleiotropy between the instrumental variables and the gut microbiota, indicating that the observed associations remained robust and were not influenced by other potential pathways. It is worth noting that similar to the case of thyroid cancer, no horizontal pleiotropy was detected in the context of instrumental variables for thyroid cancer. Leave-one-out analysis further supported the robustness of our findings, as no individual SNP significantly influenced the observed associations between the gut microbiota and TC (Figs. 5 and 6). Similarly, no individual SNP significantly affects the impact of TC on the gut microbiota composition (Fig. 7).

## Discussion

In this study, we conducted bidirectional MR analysis to investigate the potential causal relationship between gut microbiota composition and TC. Our findings revealed mutual associations between gut microbiota and TC. On one hand, we observed specific bacterial taxa associated with an increased or decreased risk of TC. For instance, the genus Subdoligranulum and class Betaproteobacteria were identified as risk factors and protective factors for TC, respectively. These findings suggest that certain microbial communities within the gut may contribute to the development or prevention of TC. The identification of these specific taxa provides potential targets for further research and therapeutic interventions. Conversely, while recent studies have predominantly explored the impact of gut microbiota on thyroid gland activity, there is a scarcity of research addressing the reciprocal influence of TC on the gut. We observed alterations in the composition of the gut microbiota following the occurrence of TC. For instance, the relative abundance of was decreased, and certain taxonomic groups, including family Defluviitaleaceae, genus Ruminococcus gauvreauii group, genus Coprobacter, genus Defluviitaleaceae UCG011, genus Family XIII UCG001, and genus Prevotella9, exhibited decreased abundance following TC occurrence, suggesting that TC may exert a regulatory influence on the gut microbiota composition.

The gut microbiota has emerged as a pivotal player in a spectrum of physiological and pathological processes, encompassing immune regulation, metabolic modulation, and disease progression. The potential influence of gut microbiota on immune responses and the metabolism of trace nutrients suggests a potential role in the regulation of thyroid homeostasis (Knezevic et al. 2020; Docimo et al. 2020; Samimi and Haghpanah 2020). Disturbances in the gut microbiota have been implicated in various thyroid disorders, including primary hypothyroidism, autoimmune thyroid disorders (AITD), and thyroid cancer (Su et al. 2020; Zheng et al. 2023; Köhling et al. 2017). With advancements in genetic sequencing technologies, researchers have delved deeper into the realm of gut microbiota. Current research on the interplay between thyroid cancer and gut microbiota predominantly focuses on clinical correlations and functional insights, which to some extent resonate with our study's findings. For instance, Zhang et al. noted that the gut microbiota profile of the thyroid cancer group featured dominance of Prevotella, Roseobacter, Coccidioides faecalis, Anaerobacter, Ruminalococcus, Neisseria, Streptococcus, and Porphyromonas, in contrast to the dominance of Mycobacterium avium, Sutterella, and Butyricimonas in the healthy control group (Zhang et al. 2019). Feng et al. observed a conspicuous enrichment of Firmicutes and Bacteroidetes in the gut microbiota of thyroid cancer patients, with the healthy cohort demonstrating an enrichment of Actinobacteria. The comparison revealed six distinct genera, including Lactobacillus, Prevotella, Roseobacter, Actinomyces, Fusobacterium, and Christensenella, exhibiting notable differences between thyroid cancer patients and healthy individuals (Feng et al. 2019). Likewise, Yu et al. unveiled elevated levels of Prevotella, Clostridium, and Spirochaetaceae, alongside decreased levels of Propionibacterium, Bacteroides, Bacteroidetes, and Firmicutes in thyroid cancer patients (Yu et al. 2022). Furthermore, an investigation targeting patients who underwent thyroidectomy for papillary thyroid carcinoma, followed by postoperative radioiodine therapy and consequent hypothyroidism, disclosed a significant reduction in gut microbiota richness compared to the healthy group. This was accompanied by marked alterations in six genera, including Prevotella, Blautia, Rectalibacter, Bifidobacterium, Fusicatenibacter, and Parabacteroides.

Currently, our understanding of the potential mechanisms governing the interaction between the gut microbiota and thyroid cancer remains limited. Within the scope of this study, we have identified specific families, such as Prevotellaceae and Ruminococcaceae, that are responsible for producing short-chain fatty acids (SCFAs) like butyrate and propionate (Kircher et al. 2022). These SCFAs are renowned for their potent anti-inflammatory and anti-tumor properties (Liu et al. 2021). However, their decreased levels within the intestines of TC patients hint at a potential influence in promoting the onset and progression of TC. Moreover, specific species such as Bifidobacterium, Ruminococcus, and Ruminococcaceae exhibit the ability to convert propionate to acetate via the acetyl-CoA pathway, while Blautia species employ an alternative enzyme called anaerobic acetyl-CoA synthetase to synthesize acetate. These intricate processes play a pivotal role in maintaining intestinal homeostasis and potentially influence the development of interconnected disorders like immune dysregulation and tumor progression (Louis et al. 2014). Concurrently, existing studies' functional enrichment analysis of the gut microbiota sheds light on the potential impact of various metabolites, including lipids, flavonoids, and phenols, in influencing the onset and progression of thyroid cancer, aligning with findings from previous research (Feng et al. 2019). Notably, our study revealed Christensenellaceae as a risk factor. In a similar vein, Lu et al. conducted comprehensive analyses involving 16S rRNA gene sequencing and LC–MS techniques on TC patient samples. Their findings uncovered a substantial reduction in the abundance of g_Christensenellaceae_R-7_group and other genera closely linked to lipid metabolism within the TC group (Lu et al. 2022). Interestingly, a metabolite known as 27-hydroxycholesterol (27HC), intricately connected with lipid metabolism, displayed diminished levels in the TC group. Earlier research has indicated the potential of cholesterol and 27HC to heighten thyroid cancer aggressiveness (Revilla et al. 2019). A recent revelation indicates a noteworthy correlation between 27HC and metabolism-associated microorganisms, notably the g_Christensenellaceae_R7_group, within the intricate network of microbial-metabolite interactions. This suggests that 27HC could potentially foster thyroid cancer proliferation driven by estrogen receptors. Concurrently, the g_Christensenellaceae_R7_group, as a central genus in the diminished genus cluster, might significantly contribute to lipid metabolic equilibrium through 27HC. Moving forward, a deeper exploration into the intricate relationship among g_Christensenellaceae_R-7_group, 27HC, and thyroid tumorigenesis holds promise and warrants further investigation.

The strengths of our study lie in the use of MR analysis, which provides a powerful tool to assess causality and overcome some of the limitations of observational studies. By leveraging genetic variants as instrumental variables, we minimized the potential for confounding and reverse causation, providing more robust evidence for the causal relationship between gut microbiota and TC. However, several limitations should be acknowledged. Firstly, our study relied on summary data from large-scale GWASs, which may introduce potential biases and limitations inherent to the original studies. Secondly, our findings were based on populations of European ethnicity, and generalizability to other populations should be interpreted with caution. Future studies incorporating diverse ethnic populations are needed to validate our findings. Lastly, while our study identified associations and potential causal effects, the exact mechanisms underlying the observed relationships remain to be elucidated. Further mechanistic studies, including functional experiments and microbiota profiling, are warranted to provide deeper insights into the gut microbiota-TC interplay.

In conclusion, our bidirectional MR analysis provides evidence supporting a reciprocal relationship between gut microbiota composition and TC. Our findings underscore the potential involvement of the gut microbiota in the development of TC. Further investigations are essential to validate our results, delve into the functional implications of distinct microbial taxa, and unravel the mechanistic connections between the gut microbiota and TC. Ultimately, a better understanding of the gut microbiota-TC interaction may open up new avenues for personalized approaches to TC management and pave the way for the development of microbiota-based therapeutics in the future.

## Supplementary Information

Below is the link to the electronic supplementary material.

Supplementary file1 (XLS 256 KB)

Supplementary file2 (XLS 10 KB)

Supplementary file3 (XLS 10 KB)

Supplementary file4 (XLS 12 KB)

## Data Availability

The original contributions introduced in this study have been incorporated within the article and Supplementary Material for reference. Additional inquiries can be directed to the corresponding author upon reasonable request.
